# ADaCGH: A Parallelized Web-Based Application and R Package for the Analysis of aCGH Data

**DOI:** 10.1371/journal.pone.0000737

**Published:** 2007-08-15

**Authors:** Ramón Díaz-Uriarte, Oscar M. Rueda

**Affiliations:** Structural Biology and Biocomputing Programme, Spanish National Cancer Center, Madrid, Spain; National Cancer Institute at Frederick, United States of America

## Abstract

**Background:**

Copy number alterations (CNAs) in genomic DNA have been associated with complex human diseases, including cancer. One of the most common techniques to detect CNAs is array-based comparative genomic hybridization (aCGH). The availability of aCGH platforms and the need for identification of CNAs has resulted in a wealth of methodological studies.

**Methodology/Principal Findings:**

ADaCGH is an R package and a web-based application for the analysis of aCGH data. It implements eight methods for detection of CNAs, gains and losses of genomic DNA, including all of the best performing ones from two recent reviews (CBS, GLAD, CGHseg, HMM). For improved speed, we use parallel computing (via MPI). Additional information (GO terms, PubMed citations, KEGG and Reactome pathways) is available for individual genes, and for sets of genes with altered copy numbers.

**Conclusions/Significance:**

ADaCGH represents a qualitative increase in the standards of these types of applications: a) all of the best performing algorithms are included, not just one or two; b) we do not limit ourselves to providing a thin layer of CGI on top of existing BioConductor packages, but instead carefully use parallelization, examining different schemes, and are able to achieve significant decreases in user waiting time (factors up to 45×); c) we have added functionality not currently available in some methods, to adapt to recent recommendations (e.g., merging of segmentation results in wavelet-based and CGHseg algorithms); d) we incorporate redundancy, fault-tolerance and checkpointing, which are unique among web-based, parallelized applications; e) all of the code is available under open source licenses, allowing to build upon, copy, and adapt our code for other software projects.

## Introduction

Copy number alterations (CNAs) in genomic DNA have been associated with complex human diseases, including cancer [1]–[7]. For instance, amplification of oncogenes is one possible mechanism for tumor activation [8], [9]. Patient survival and metastasis development have been shown to be associated with certain CNAs [1]–[7] and, by relating patterns of CNAs with survival, gene expression, and disease status, studies about copy number changes have been instrumental for identifying relevant genes for cancer development and patient classification [1], [2], [10]. One of the most common techniques to detect CNAs is array-based comparative genomic hybridization (aCGH), a term that includes platforms such as ROMA, oaCGH (including Agilent, NimbleGen, and many non-commercial, in-house oligonucleotide arrays), BAC, and cDNA arrays [1], [11] (see section “Program overview” for comments on Affymetrix SNP arrays). The availability of aCGH platforms and the need for identification of CNAs has resulted in a wealth of methodological studies (see reviews in [12], [13]). Associated with this statistical work, several tools have been developed for the analysis of aCGH data, but these tools fail minimal requirements for both end-users and bioinformaticians/biostatisticians. Thus, we have developed ADaCGH.

An ideal tool for the analysis of aCGH data should allow the user to choose among several of the best performing algorithms (e.g., see comparative reviews of [12], [13]). For end-users, the suitability of web-based applications for aCGH data analysis has been emphasized before (e.g., [14], [15]), and web-based tools do not require software installation by the user, nor concerns about hardware [16]. Moreover, web-based applications ease the linking of the results from aCGH analysis to external databases (e.g., Gene Ontology, PubMed) and, thus, ultimately, ease the biological interpretation of the results. Moreover, web-based applications can use parallel computing, leading to impressive decreases in users' waiting time. Finally, the source code of such a tool should be freely available under an open source license: it allows other researchers to extend the methods, provide improvements and bug fixes, and verify claims made by method developers, and ensures that the international research community remains the owner of the tools it needs to carry out its work [17], [18].

## Results

### Program overview

ADaCGH is available both as a web-based application and as an R package. The statistical and graphical functionality is provided by the R package, which implements parallelized versions of all algorithms. Thus, both the R application and the web-based application can take advantage of multicore processors and clusters of workstations. ADaCGH uses eight algorithms for CNA detection, including the best performing ones from recent reviews [12], [13]. The web-based application is available at http://adacgh2.bioinfo.cnio.es. The source code for both the web-based application and the R package are available from both Launchpad (http://launchpad.net/adacgh) and Bioinformatics.org (http://bioinformatics.org/asterias/bzr/adacgh). The R package is also available from CRAN (http://cran.r-project.org/src/contrib/Descriptions/ADaCGH.html). Documentation and examples for the web-based application are available from http://adacgh2.bioinfo.cnio.es/help/adacgh-help.html. Documentation for the R functions are available as in any standard R package.

Input for the web-based application are text files with aCGH data and location information. The aCGH data are often log ratios from array-based CGH platforms (the base of the logarithm is not of great importance, but base 2 logs are often of simpler interpretation). Affymetrix SNP data can also be analyzed, but external preliminary steps are required, as is common with Affymetrix SNP data, that allow to go from the MM and PM data (and, possibly, information on GC content and fragment length) to numerical values that play a role similar to the log ratios of aCGH arrays (for examples see [19]–[24]). Further details are provided in the help page for the web-based application http://adacgh2.bioinfo.cnio.es/help/adacgh-help.html#input.

The output (oth the web-based and R-package versions) are text files with the segmentation results and figures. The figures allow for genome-wide views and chromosome-wide views, array-by-array views and collapsed views over arrays. Figures include clickable links to our application IDClight (http://idclight.bioinfo.cnio.es) [25] which provides additional information, including mapping between gene and protein identifiers, PubMed references, Gene Ontology terms, Kegg and Reactome pathways for genes. In addition, the web-based application allows for sending the sets of genes showing gain, loss, and CNA (gain or loss) to our tool PaLS (http://idclight.bioinfo.cnio.es) to examine PubMed references, Gene Ontology terms, KEGG pathways or Reactome pathways that are common to a user-selected percentage of genes. When the arrays correspond to human samples, we provide links to the Toronto Database of Genomic Variants (http://projects.tcag.ca/variation/) in the chromosome-wide plots.

### Benchmarks

**Figure 1:**
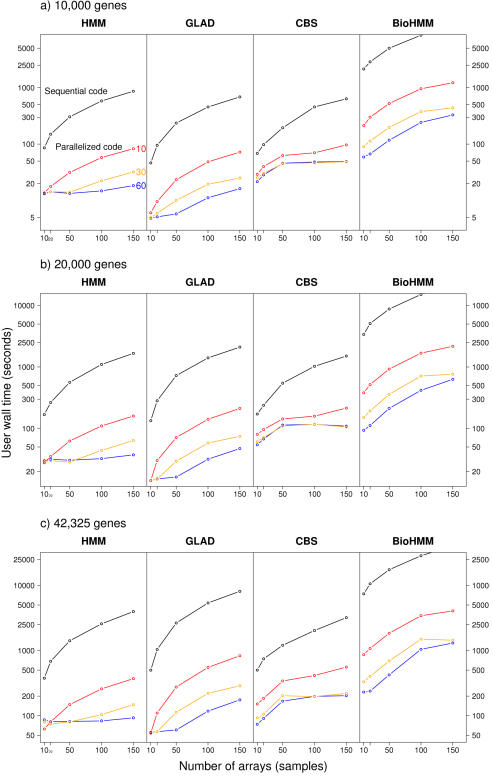
Effects of parallelization of the R code on the user wall time for several methods. Values shown are the mean of four replicates, obtained in an otherwise idle cluster with 30 nodes, each with two dual-core AMD Opteron 2.2 GHz CPUs and 6 GB RAM, running Debian GNU/Linux and a stock 2.6.8 kernel, with version 7.1.2 of LAM/MPI and version 2.1.4 (patched) of R. Numbers next to the lines (60, 30, 10) indicate the total number of Rslaves in the cluster (2 slaves per node, and a maximum of 30 nodes used).

Speedups achieved by parallelizing the R code are shown in Figure 1 for four popular methods. The speedups range from 40× to 45× (GLAD, HMM), to 30× (BioHMM) and 15× (CBS).

**Figure 2:**
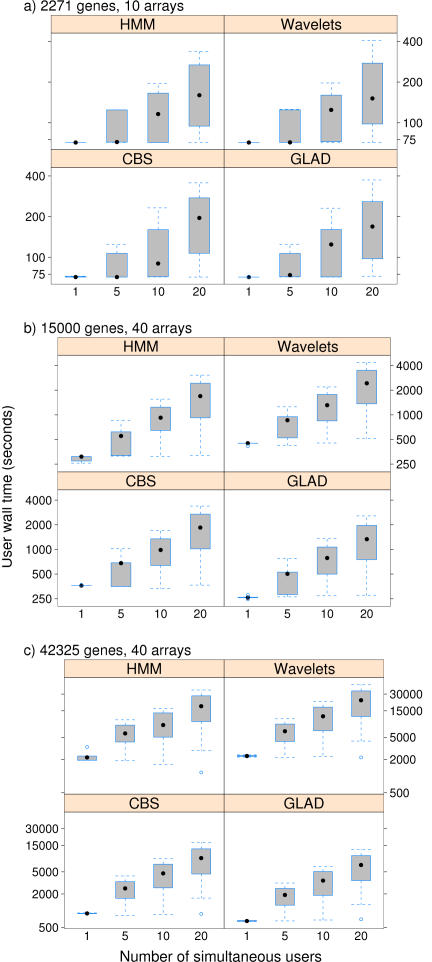
User wall time of the web-based application as a function of simultaneous users. To increase the realism of simultaneous accesses, there is delay of 5 seconds between simultaneous accesses, as might be expected, for example, from a classroom demonstration (i.e., when simulating 20 simultaneous users, the cluster is actually receiving new connections over a 20 * 5 second period, with one new connection every 5 seconds). Values shown are the mean of several runs: 5 for 1 user, 5 for 5 users, 10 for 10 users, and 20 for 20 users. Hardware and software the same as in Figure 1.

Figure 2 shows user wall time of the web-based application as a function of the number of simultaneous users using the application in that very moment. ADaCGH can handle a large number of simultaneous users as a result of both parallelization of the computations and load balancing of the non-parallelized code. Increasing the number of users from 1 to 5 has a minor effect in the mean user wall time. Increasing the number of users above 5, however, has a linear effect in the mean user wall time. This is the result of the limits we have set to prevent any one node from swapping to disk (swapping would occur if we run too many simultaneous process with a large memory consumption). Situations with 5 or more simultaneous users are unrealistic, since the average number of daily users of ADaCGH is less than 6. The above benchmarks, nevertheless, show that ADaCGH can handle even those high numbers of users, which makes it suitable for classroom and demonstration use.

## Discussion

Our main foci when developing ADaCGH have been:

- Implementing all of the best currently available algorithms/methods. Applications targeted to biomedical researchers should include several of the best methods to assure the user availability of choices and the possibility of using more than one method on the same data set.
  We have implemented all of the best performing methods for the analysis of aCGH data, based on [12], [13], plus several others that can be of interest. Moreover, we have extended some methods (e.g., using merging of segmentation results in both the wavelet-based smoothing and CGHseg) to accommodate the latest recommendations [12] and needs in the field (e.g., mapping to gain/loss/no-change to allow interpretation based on type of alteration).
- Taking user waiting time seriously. For web-based applications it is not enough to simply provide a thin wrapper of CGI code that can never be faster than the original BioConductor package.
  We have parallelized all of the algorithms, some of them in several different ways (e.g., at the arrays or at the arrays by chromosomes level). The major opportunities for significant performance gains and ability to handle large datasets lie in the increasing availability of multicore processors and clusters built with off-the-shelf components [26]–[29], as the rate of increase in processor speed has slowed down significantly in the last five years. In our application, parallelization's benefits are: a) significant decreases in user wall time; b) examples for parallelization of further algorithms; c) speed increases that will allow researchers to conduct comprehensive comparative studies among methods in reasonable time.
- Making the complete code (including algorithms and the web-based application) available as open source.
  Our complete repositories are available. Licenses used are GNU GPL for the R package (required for compatibility with the R and BioConductor packages used) and the Affero Public license for the rest of the code. The later ensures that the research community remains the owner of the web-based and fault-tolerant logic, and that any modifications for usage in other web-based applications will also be owned by the research community. Moreover, we have tried to incorporate standard best practices in software development (see review and references in [30]) and the usual open source development mode [31] to allow for the building of a community of contributors. Finally, of the existing aCGH applications we are the only ones to provide extensive functional and regression testing.
- Providing an example that be used as a model for related projects, significantly decreasing development time of other applications.
  We have avoided the usage of Python-specific web frameworks, so that the logic of the application can be translated to any other language. We have also avoided R-specific extensions as a server or web-based application, so the model can be imitated with other computational engines (e.g., code written in C).

Several tools are available for the analysis of aCGH data. The majority of the available ones are summarized in the recent paper by [15]. Since then, a few others have appeared: arrayCGHbase [32], CGHScan [33], CAPweb [14], and ISACGH [34]. Of those 29 applications, only seven (or eight) implement one of the methods with good performance in [12], [13]. The other 22 (or 21) provide no formal segmentation method, or implement approaches that are either ad-hoc (e.g., most of the simple thresholding methods) or have not been carefully compared with other methods. Thus, only a handful of the implemented methods are really of direct, immediate interest for end users. Of the remaining applications, three are BioConductor R packages (aCGH, DNAcopy, GLAD) that implement only a single method and are, of course, not web-based applications. These packages are extremely important for biostaticians and bioinformaticians (e.g., these three packages are used by ADaCGH) but are not particularly user-friendly. Of the remaining five, CNAG [35] fits only one type of model (HMM) and only to oligo-based arrays. dCHIP [36] implements a type of HMM that requires reference samples and, again, is only one specific type of model. CGHExplorer [37] implements only the ACE approach. CGHPRO [38] includes both the HMM of Fridlyand [39] and CBS [40], by using the BioConductor packages aCGH and DNAcopy. Their program is tied to specific software (e.g., the user needs to install mysql) and databases (build from April 2003 of the UCSC Genome Browser). Moreover CGHPRO is bound in speed by the speed of the DNAcopy and aCGH BioConductor packages and incorporates none of the computational advantages of ADaCGH, and it is not web based. ISACGH [34] is a web-based application that includes GLAD and CBS but, as before, its speed is bound by the speed of the DNAcopy and GLAD BioConductor packages and incorporates none of the computational advantages of ADaCGH; moreover, the source code is not available. Finally, CAPweb [14] is tied to just one specific method (GLAD), again making it difficult to compare the outcome from several different well-performing algorithms, and does not provide complete source code.

In summary, ADaCGH is a unique application from the end user's standpoint: all of the best performing algorithms are accessible and, as it uses parallelization, it provides much faster execution than the original R packages. ADaCGH is also a unique application for methodological reasons. It provides the complete source code of the only application that combines parallel computing with a web-based front end, including fault tolerance and checkpointing, and extensive functional and numerical testing. In conclusion, ADaCGH sets a much higher standard than any of the previous applications for the analysis of aCGH.

## Methods

### Algorithms: implementation and additions

Most of the segmentation algorithms included in ADaCGH are available, in sequential versions, from R or BioConductor packages. For Circular BinarySegmentation [40] we use the BioConductor package “DNAcopy”; for the (homogeneous) Hidden Markov Models [39], aCGH; for the non–homogeneous Hidden Markov Models in [41] we use BioHMM; PSW (SWARRAY in the original paper [42]) uses the cgh package; kernel non-parametric smoothing in GLAD [43] uses the GLAD package. For wavelet-based smoothing [44] we have used R code kindly provided by their authors, L. Hsu and D. Grove. The Gaussian process model in CGHseg [45] uses functions implemented in the package tilingArray; we have, however, implemented the original author's adaptive penalization approach (the tilingArray and snapCGH BioConductor packages use as possible penalization BIC or AIC, but not the adaptive one recommended by Picard et al. [45]). For Analysis of Copy Errors [37] we use C code written by us based on the original Java code, and called from R.

For merging segmentation results, to map the segmented output to “gain/loss/no-change” states, we use either the original procedure of the authors, as in GLAD, or the procedures examined in [12] for CBS and HMM, implemented in the mergeLevels function of the aCGH package.

For the wavelet-based approach [44] we have adapted the mergeLevels approach. The original paper [44] does not map the segmentation results to a set of ”gain/loss/no-change” levels. We have followed the same approach as in CBS, and use here the mergeLevels procedure. It must be emphasized that this is an experimental procedure, not described in the original paper. Moreover, the wavelet-smoothing procedure returns smoothed values that rarely fall into a set of categories, so applying mergeLevels here often leads to non-sense results. Thus, we apply mergeLevels after running the original clustering procedure of this method with a very small threshold for merging (currently set to 0.05, or five times smaller than the default of 0.25); some preliminary trials show that the final outcome from mergeLevels is not sensitive to small variations around this threshold.

The original paper on CGHseg [45] includes no details on mapping the segmentation results to the ”gain/loss/no-change” levels. We thus use mergeLevels on the output. With this approach, CGHseg is one of the best overall performers (on par with Circular Binary Segmentation) in our comparison of several methods for aCGH analysis (see Supplementary Material to [46]) using the complete simulated data set in [12]. An alternative, naive mapping approach (setting the most abundant class to the “no-change” level, and all others to gain or loss depending on their mean), leads to much worse performance (see Supplementary Material of [46] for details).

For finding minimal common regions of gains and losses we use the procedure in [5] as implemented in the cghMCR BioConductor package.

Where appropriate, we have re-written some of the above code for parallelization (see below). Parallelization uses the Rmpi (http://www.stats.uwo.ca/faculty/yu/Rmpi) and papply (http://ace.acadiau.ca/math/ACMMaC/software/papply/) R packages by H. Yu and D. Currie, respectively.

Clickable figures are generated from the R code with some additional calls to Python code. In the web-based application, Python is used for CGI, initial data validation, and to ensure proper seting-up and closing of the parallel infrastructure (booting and halting the LAM/MPI universes).

### Algorithms: Parallelization

Parallelization of algorithms has been carried out to maximize speed gains from the distribution of the computation (see [47], [48] for general guidelines), while making further extensions and applications to other methods as easy as possible, requiring only writing some wrapper code to existing segmentation code. For the aCGH algorithms considered, there are embarrassingly parallelizable computations at the chromosome by array level. Alternatively, we might parallelize at the array level, looping (sequentially) over chromosomes, or parallelize at the chromosome level, looping (sequentially) over arrays, with the later option only being reasonable for the ACE algorithm. In contrast to parallelizing at the array level, parallelizing at the array by chromosome level can use all available CPUs when there are few arrays. However, parallelizing at the array by chromosome level might not always be appropriate: the tasks are of very uneven duration (e.g., segmenting chromosome 1 vs. segmenting chromosome 21), much more communication is needed between the master and the slaves and, when there is merging (as in CBS, HMM, BioHMM, and our implementations of CGHseg and wavelet smoothing) synchronization barriers are needed before merging can be performed (where the merging algorithm would be parallelized at the only possible level, which is array).

**Figure 3:**
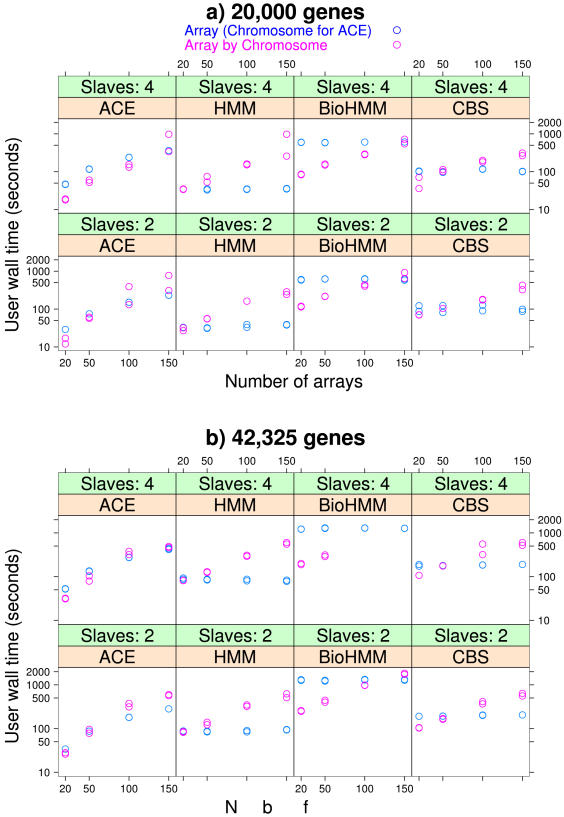
Comparing parallelization schemes. User wall time of the R code using parallelization over arrays by chromosomes or over array (all methods shown, except ACE) or chromosome (ACE). “Slaves: 2” or “Slaves: 4” indicates the number of slaves per node. The two timings shown were obtained from an otherwise idle cluster, with hardware and software as in previous figures.

To choose the best parallelization scheme, we have examined the alternatives where this flexibility was easily available, taking into account different numbers of genes per array, different numbers of slaves per node, and different numbers of arrays. For HMM, BioHMM, and CBS we have compared parallelization at the array by chromosome vs. at the array level, and for ACE we have compared parallelization at the array by chromosome vs. at the chromosome level. Results are shown in Figure 3. In most cases, parallelization at the array level is better (it results in smaller users' wall time). Only for small number of arrays (i.e., when many of the CPUs are idle if parallelization is at the array level) can parallelization at the array by chromosome level perform better, as we would expect from the trade-offs involved (see above). We have used the results from this figure to automatically choose the parallelization level used in any given run. Of course, the optimal parallelization is strongly dependent on the underlying hardware, mainly CPU number and speed, number of cores, caches' sizes, and network speed.

For the current code, the execution of HMM, BioHMM, CBS, and ACE is parallelized at the array (chromosome if ACE) or array by chromosome level depending on the number of arrays. Following the main results with HMM and CBS, wavelet-based smoothing and CGHseg are parallelized at the array level. GLAD and PSW are parallelized at the array level as the code of the basic algorithms themselves would not allow for easily maintainable finer grained parallelization.

An additional concern with multicore CPUs is, for each node, whether to use as many Rmpi slaves as cores (4 in our case) or as sockets (2 in our case), as the different cores share resources that different processors do not [28]. The results of Figure 3 show that using 4 slaves per node rarely leads to performance increases but, because of increased memory usage, can prevent some processes from completing (e.g., BioHMM with 42325 genes and either 100 or 150 arrays).

Figure creation in the web-based application is parallelized at the array level, by writing to a shared directory (accessed via NFS), except for the figures where all arrays are superimposed, where parallel execution is impossible.

### Web-based application: Program logic

**Figure 4:**
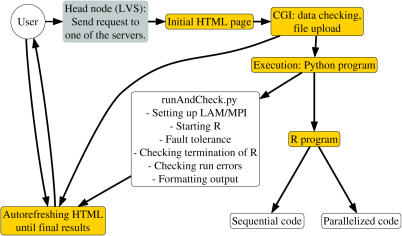
Overview of the flow of information between the main components of the web-based application.

**Figure 5:**
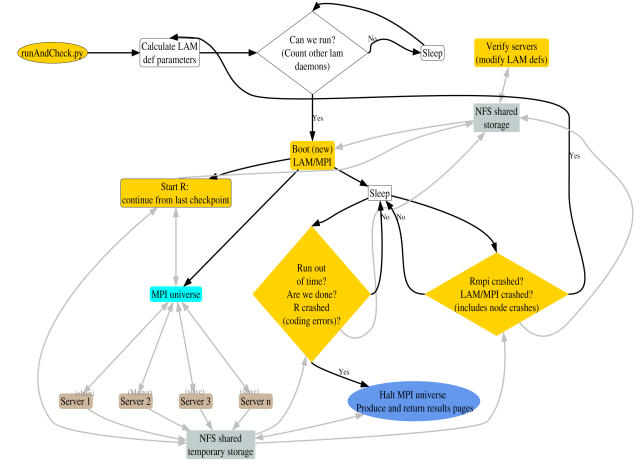
Flow of information between application components: main mechanisms for crash recovery and fault tolerance. Black: execution flow. Gray: read (←) or write (→) to/from files/nodes/hardware elements.

The main application components, their relationship, and some key hardware components are shown in Figures 4 and 5. Our installation of the web-based application runs on a cluster of 30 workstations with two dual-core AMD Opteron CPUs. The HTTP request from a user arrives at one of the two master nodes; currently, we are using Linux Virtual Server (http://www.linuxvirtualserver.org/) to provide load balancing of the web serving and redundancy (see below), but we have also used Pound (http://www.apsis.ch/pound/) and alternative mechanisms could be used. This request is sent to one of the server nodes. In there, this request returns a static HTML page, for simpler and faster execution, with the appropriate fields for file upload.

Upon hitting the “submit” button on the HTML page, a (Python) CGI is executed in the given server node. This CGI carries out basic data management and verification. Briefly, a temporary directory in a shared (via NFS) file system is created, the data files verified for basic correctness, and then stored in this temporary directory. This temporary directory has a name formed by 13 random digits plus the process ID plus the time of creation; this makes it virtually impossible that two runs of the application will write data to the same temporary directory. This CGI returns a (temporary) results HTML file to the user which is an autorefreshing HTML page (to prevent time-outs in the client-server connection) with the URL address. At the termination of the run, this temporary HTML file will be substituted by the final results file. The last job of this CGI is to spawn a Python program (identified as “runAndCheck.py” in the figures) that does the bulk of managing the MPI environment, launching R, and providing fault tolerance.

This runAndCheck.py program carries out several major tasks. First, based upon the size of the uploaded files, it determines the parameters to use for the LAM/MPI universe (the number of Rslaves that will be spawned in each node, and the maximum number of ADaCGH processes that are allowed to run simultaneously at any time). Next, it determines if a new process can be run (by counting the number of lam daemons in the node); if it cannot run yet, it waits and checks again after a specified interval. Otherwise, a new LAM/MPI universe is booted, and an R process started. runAndCheck.py is also in charge of fault tolerance and crash recovery (see below). Eventually, upon either successful or unsuccessful termination, a results HTML file is constructed, and returned to the user; this file replaces the above temporary results file.

A combination of R, Python, and Javascript code is involved in generating lists of genes for PaLS (e.g., the list of all genes that show gains in copy number) and providing figures with clickable links to our IDClight application [25].

In addition to the above major programs (the CGI and runAndCheck.py), there is a cron job that executes periodically to verify which nodes (servers) are responding and can be used by LAM/MPI. If needed, the default LAM/MPI configuration files are modified adding or deleting entries for the corresponding nodes.

### Fault tolerance and crash recovery

Partial failure is unavoidable in distributed applications [49]–[51]. We use several layers to provide fault tolerance and crash recovery. Linux Virtual Server with heartbeat and mon (http://www.linuxvirtualserver.org/docs/ha/heartbeat_mon.html) using two master nodes provides redundancy in case one of the master nodes fails, and monitors the server nodes so that no HTTP requests are sent to non-responding nodes. Results and temporary computations are stored in a shared storage space that uses RAID 50; this allows both access from nodes different from the one where computations started, and permits the cluster to continue working in case of failure of some of the disks.

The above mechanisms, however, do not offer a way to continue an ongoing calculation in case of failure. Common sources of partial failure are a crash in one of the nodes that are running a slave MPI job, MPI (or Rmpi) errors, and network problems. These problems are particularly common (and difficult to correct via a specific, immediate, human intervention) in web-based applications that have to run unattended with, ideally, 100% availability. Moreover, any of these are recoverable errors and, thus, stopping the complete calculation and returning an error message to the user (forcing the user to relaunch the process) or, worse, halting indefinitely, are suboptimal ways of responding to the above errors.

As illustrated in Figure 5, the web-based application incorporates a mechanism that, periodically, examines MPI and R logs and existing LAM/MPI daemons to determine if any of the above problems have occurred. If they have, a new LAM/MPI universe is booted (after determining which nodes are currently alive and can run MPI processes), and a new R process launched. To prevent carrying out again computationally costly calculations, the R code includes checkpoints so that calculations are not started from the beginning but only continued from the point they were stopped.

The above mechanism of fault recovery is independent of another mechanism that checks for completion. Completion can either be successful or unsuccessful. The later can be caused by errors in our R code and, in such a case, we want to abort the calculation immediately, return a message to the user, and log the problem to allow us its prompt fixing. These errors are detected via monitorization of R logs and currently running R processes. In a similar way are handled fatal errors in libraries we depend upon, such as failures in optimization that are occasionally encountered with BioHMM.

### Testing and bug tracking

ADaCGH includes a comprehensive test suite that uses FunkLoad (http://funkload.nuxeo.org). These functional tests cover the user interface and the numerical output, including verification that our parallel implementations return the same values as the original sequential ones. All the tests can be run on demand, and whenever new changes are introduced in the software, thus ensuring appropriate quality control and regression testing. The tests are available under the “ADaCGH2” directory from the repositories (http://bioinformatics.org/asterias/bzr/Testing or http://launchpad.net/functional-testing). In addition to the uses from its release date (November 2005) and the FunkLoad test suite, the code has undergone extensive usage from the benchmark results shown below. Bug-tracking is available from http://bioinformatics.org/asterias.
